# The Transcultural Adaptation and Validation of the Chinese Version of the Duke Anticoagulation Satisfaction Scale

**DOI:** 10.3389/fphar.2022.790293

**Published:** 2022-02-23

**Authors:** Yibo Wu, Shujie Dong, Xinyi Li, Haiping Xu, Xiaohui Xie

**Affiliations:** ^1^ Department of Pharmacy Administration and Clinical Pharmacy, School of Pharmaceutical Sciences, Peking University, Beijing, China; ^2^ Department of Pharmacy, Peking University Third Hospital, Beijing, China; ^3^ School of Pharmaceutical Sciences, Shandong University, Jinan, China

**Keywords:** anticoagulation, satisfaction, scale, validation, quality of life

## Abstract

**Background:** The Duke Anticoagulation Satisfaction Scale (DASS) offers a method to measure the quality of life and satisfaction of patients taking oral anticoagulants. The study aimed to validate the Chinese version of the DASS in Chinese patients on anticoagulation therapy.

**Methods:** The DASS was translated, back-translated, and transculturally adapted into the Chinese version and then administered to participants taking oral anticoagulants in a physician–pharmacist collaborative anticoagulation clinic at a tertiary teaching hospital from October 2019 to December 2020. Reliability was analyzed through Cronbach’s alpha (α) and split-half reliability. Confirmatory factor analysis was performed to test the structural validity of the scale. Exploratory factor analysis was performed for items in the scales using the varimax rotation method.

**Results:** A total of 189 patients completed the Chinese version of the DASS. Four dimensions and 23 items were included, with Cronbach’s α values of 0.89, 0.81, 0.89, and 0.74 for limitations on physical activities, diet restrictions, hassles and burdens, and positive psychological effect, respectively. Cronbach’s α coefficient of whole scale was 0.91. The split-half reliability of this scale is 0.747 (>0.7).

**Conclusion:** The Chinese version of the DASS indicated excellent reliability and validity, compared to the original version. It could provide a practical instrument for healthcare practitioners to evaluate satisfaction and quality of life for anticoagulated patients in China. The difference in quality of life between patients taking warfarin and novel oral anticoagulants (NOACs) needs to be further explored in future studies.

## Introduction

Oral anticoagulants, including warfarin and novel oral anticoagulants (NOACs), are used to treat venous thromboembolism, such as pulmonary embolism and deep vein thrombosis, and prevent stroke in patients with atrial fibrillation (Keeling et al., 2011; Mueck et al., 2014; Cohen et al., 2015; Cohen et al., 2016; Witt et al., 2016; Bai et al., 2017). If used improperly, oral anticoagulants can result in thrombosis or bleeding (Lin et al., 2019; Shields et al., 2019) and therefore reduce the quality of life (Lozano Sanchez and Areitio-Aurtena Bolumburu, 2008; Sallinen et al., 2019). Dissatisfaction with anticoagulation is associated with poor adherence, international normalized ratio (INR) control, and clinical outcomes (Perino et al., 2019). Satisfaction with anticoagulant therapy can substantially improve patients’ quality of life (Benzimra et al., 2018).

Common generic scales are widely used to measure health-related quality of life, including the EuroQol Five Dimensions Questionnaire (EQ-5D) (Balestroni and Bertolotti, 2012) and the MOS item short form health survey (SF-36) (Ware and Sherbourne, 1992). However, their comprehensiveness hinders the deep understanding of patients on specific medications. Condition-specific scales are intended to narrowly focus on detailed aspects of health-related quality of life that are of the greatest importance for that condition. The Duke Anticoagulation Satisfaction Scale (DASS) is specially developed and validated to measure the quality of life and satisfaction of patients taking oral anticoagulants (Samsa et al., 2004). Four dimensions (limitations on physical activities, dietary restrictions, hassles and burdens, and positive psychological impacts) and 25 items in the original scale were replaced by three dimensions (limitations, hassles and burdens, and positive impacts) and 25 items according to the results of the factor analysis (Samsa et al., 2004). DASS was adapted to the Brazilian Portuguese version (Pelegrino et al., 2012), Arabic version (AlAmmari et al., 2020), and Maltese version (Riva et al., 2019), all of which showed satisfactory psychometric proprieties. To the best of our knowledge, no literature related to the Chinese version of the scale has been reported. Considering the large Chinese population and obvious differences in diet, living habits, medical-seeking behaviors, culture, and psychology, the DASS needs to be validated in Chinese patients.

Therefore, to facilitate the evaluation of quality of life and address the gap of DASS applied in Chinese, this study aimed to assess the reliability and validity of the Chinese version of the DASS among patients receiving oral anticoagulant therapy.

## Materials and Methods

### Design and Participants

This study was carried out in a physician–pharmacist collaborative anticoagulation clinic at a tertiary teaching hospital from October 2019 to December 2020. We included patients if they 1) had been diagnosed with venous thromboembolism or atrial fibrillation, or stroke, and needed to take oral anticoagulants for a long time; 2) took oral anticoagulants including warfarin and NOACs; 3) were older than 18 years. The exclusion criteria were as follows: 1) malignant tumors; 2) severe organ failure; 3) history of psychiatric disease, cognitive impairment, or organic brain disorders, and inability to understand and answer questions; and 4) discontinuation of anticoagulation therapy or loss to follow-up due to any cause. All above mentioned records were collected through a medical record system. Basic information of participants was collected, including sociodemographic and clinical characteristics (e.g., gender, age, marriage status, living alone or not, education, history of thrombosis event, history of stroke, health insurance scheme, per capita family monthly income, frequency of physical exercise, and people responsible for medication) and anticoagulation therapy (e.g., type of medication and duration of medication).

### Translation, Back-Translation, and Transcultural Adaptation of DASS

Permission to translate and adapt the DASS was obtained from Dr. Samsa, the author of the original version, by email. The English version of the DASS was translated, back-translated, and transculturally adapted into the first draft Chinese version following the method of Guillemin et al. (1993).

#### Translation and Back-Translation

Step 1: Forward translation: Translation was carried out independently by two native Chinese speakers fluent in English to form the first Chinese version of the DASS. Translator 1, a master’s student of pharmacy who knows medical terms well, adapted the scale from a clinical perspective, ensuring equivalence between the translated scale and the original one. Translator 2, a master’s student of English major without a medical background, translated from a language perspective in efforts to reflect the language habits of the public.
Step 2: Integration: Another master’s student of pharmacy, who is a native Chinese speaker fluent in English and was not involved in “forward translation,” conducted a comparative analysis of the two translations. The three researchers discussed divergences and coordinated with each other to produce the second Chinese version of the DASS. The integration of differences in this phase was generated on the unanimous agreement of the three researchers.
Step 3: Back-Translation: Two native Chinese-speaking and perfect bilingual translators back-translated the second Chinese version into the back-translated version. Neither of the two back-translators was aware of the original version or had a medical background, which avoided information bias and helped find latent translation inconsistencies. Finally, a master student of pharmacy who was a Chinese native speaker, fluent in English, and not involved in translation or back-translation, as a coordinator, compared the back-translated version with the original version and made some modifications, based on which back-translators translated again until it was in harmony with the original English version. Thus, we obtained the first draft Chinese version.

#### Transcultural Adaptation

Some items in the scale were modified to adapt to the Chinese cultural background, which was called cultural adaptation. 1) Expert consultation: an expert committee composed of 2 anticoagulation pharmacists, 2 cardiovascular physicians, 2 nurses specialized in care delivery to patients using oral anticoagulation, 1 English expert, 1 nurse researcher experienced in cultural adaptation and validation studies about quality of life instruments, and all translators, including forward translators, integrating translators, and back translators, reviewed and adapted each item in the first draft Chinese version on semantic, idiomatic, experiential, and conceptual equivalence. A prefinal version was generated after adaptation and modification according to Chinese culture and language habits. 2) Pretest: Thirty patients on oral anticoagulation who were Chinese native speakers and eligible for the inclusion criteria meeting item screening, reliability, and validity evaluation were included in the pretest. The scales were handed out after explaining the purpose of the study and obtaining informed consent. Then, interviews were conducted to ask if the scale contained ambiguous, incomprehensible, or disagreeable items. The final Chinese version of the DASS was completed after correction and proofreading for prefinal revision of the scale according to consistent feedback from interviews.

### Item Screening, Reliability, and Validity Evaluation

#### Questionnaire Design

The questionnaire consists of two parts, namely, a general information questionnaire and the Chinese version of the DASS. 1) General information questionnaire: gender, age, education, retirement status, marriage status, living alone or not, health insurance scheme, body mass index (BMI), and frequency of physical exercise. 2) Chinese version of the DASS: It included 25 items distributed in four dimensions including limitations on physical activities, diet restrictions, hassles and burdens, and positive psychological effect. Five-point Likert scale was applied: not at all = 5, a little = 4, moderately = 3, a lot = 2, very much = 1. If the item was not applicable, then the respondent was asked to choose “not at all.” Questions 3h, 4a, 4b, 4f, 4h, and 4j were reversely coded. The higher scores indicate greater satisfaction with the use of oral anticoagulants, less hassles, less burdens, and smaller psychological impact.

#### Data Collection

Questionnaires were conducted face-to-face or via telephone based on convenience sampling from October 2019 to December 2020. Interviewers obtained informed consent during the survey. Most patients completed the questionnaires by themselves. For those who had visual deficits, mobility disability, or traffic restrictions, researchers assisted them by expressing questions and answers orally with no suggestibility.

#### Statistical Methods

Data were analyzed using SPSS/WIN 22.0 (IBM Corp., Armonk, NY, United States) and AMOS 23.0 (IBM Corp., Armonk, NY, United States). If Kaiser–Meyer–Olkin (KMO) > 0.5 and Bartlett spherical test yielded *p* < .05, the data were suitable for factor analysis. Exploratory factor analysis was performed for items in the scales using the varimax rotation method. A cumulative contribution rate >50% is an acceptable range, and >70% is a good range. Items whose selection rate was higher than 80% and coefficient of variation (CV) value was lower than 0.2 were removed. For some items whose absolute t values were obtained by using the independent sample *t* test in a high-score group (highest 27%) and a low-score group (lowest 27%), they were deleted if absolute t values were both lower than 3. Moreover, item correlations with a coefficient *r* < 0.4 and *p* > .5 related to the total score of the scale were also expurgated. If Cronbach’s α coefficient of the dimension to which an item belongs increases significantly after its deletion, it means that the item will reduce the inner correlation and should be deleted.

Cronbach’s α coefficient and split-half reliability were used to test the scale reliability, with a value ≥ 0.70 considered good reliability (Webb et al., 2006). Confirmatory factor analysis using AMOS 23.0 was performed to test the structural validity of the scale. Fit indices included chi-squared over degrees of freedom (CMIN/DF) (values < 3 indicated a good fit) (Hu and Bentler, 1999), goodness-of-fit index (GFI) (values > 0.85 indicated an acceptable fit) (McDonald and Ho, 2002), comparative fit indices (CFI) (values > 0.9 indicated an acceptable fit) (McDonald and Ho, 2002), root-mean-square error of approximation (RMSEA) (values < 0.08 indicated an acceptable fit) (McDonald and Ho 2002), normed fit index (NFI) (values > 0.9 indicated a good fit) (Bentler and Bonett, 1980), and the Tucker–Lewis index (TLI) (values >0.9 indicated an acceptable fit) (Hu and Bentler, 1999).

## Results

### Translation, Back-Translation, and Transcultural Adaptation of DASS

The original questionnaire of the Chinese DASS was formulated through translation, back-translation, and cultural adaptation. In addition, some of the statements have been modified, such as “chiropractor” was modified to “masseur”. Four dimensions (limitations on physical activities, dietary restrictions, hassles and burdens, and positive psychological impacts) and 25 items were included in this questionnaire. Response options were on a 5-point scale that indicated how much the items influence the respondent. The items and their expression are illustrated in Supplementary Table S1.

### Subjects

Among 189 respondents, more than half of the respondents were male (61.4%). A total of 67.2% of respondents were on NOAC therapy, while 32.8% were on warfarin therapy. A total of 23.3% of respondents had an indication for thrombosis events, with 64.6% for atrial fibrillation and 13.2% for stroke. The sociodemographic and clinical characteristics of all respondents after starting oral anticoagulants (OAC) therapy are shown in Table 1. Among patients taking NOACs, 56 (29.6%) were on rivaroxaban and 71 (37.6%) were on dabigatran. None was using edoxaban or apixaban since edoxaban was not covered by medical insurance and apixaban is only indicated for venous thromboembolism prophylaxis in China.

**TABLE 1:** Sociodemographic and clinical characteristics of the subjects.

Characteristic	Number
Sex, *n* (%)	
Male	116 (61.4%)
Female	73 (38.6%)
Age (years), *n* (%)	
Less than 60	55 (29.1%)
61–70	66 (34.9%)
More than 70	68 (36.0%)
Marriage status, *n* (%)	
Unmarried, divorced, or widowed	10 (5.3%)
Married	179 (94.7%)
Living alone or not, *n* (%)	
Yes	14 (7.4%)
No	175 (92.6%)
Education, *n* (%)	
Junior high school and below	83 (43.9%)
High school and technical secondary school	61 (32.3%)
University degree, or above	45 (23.8%)
Health insurance scheme, *n* (%)	
Urban Resident Basic Medical Insurance	35 (18.5%)
New Rural Cooperative Medical Scheme	29 (15.3%)
Urban Employee Basic Medical Insurance	93 (49.2%)
Self-paying	32 (16.9%)
Frequency of physical exercise, *n* (%)	
Almost everyday	124 (65.6%)
3 to 4 times a week	39 (20.6%)
1–2 times a week or less	26 (13.8%)
Anticoagulant drugs, *n* (%)	
Warfarin	62 (32.8%)
Rivaroxaban	56 (29.6%)
Dabigatran	71 (37.6%)
Indication of oral anticoagulation, *n* (%)	
Atrial fibrillation	122 (64.6%)
Venous Thromboembolism	44 (23.3%)
Stroke	25 (13.2%)
People responsible for medication, *n* (%)	
Family members	19 (10.1%)
Oneself	170 (89.9%)
Duration of anticoagulation, *n* (%)	
Less than 6 months	90 (47.6%)
6 months to 1 year	43 (22.8%)
More than 1 year	56 (29.6%)

### Item Screening

#### Exploratory Factor Analysis

The KMO value of the original questionnaire was 0.94, and Bartlett sphericity tested a statistically significant value (*p* < .001), indicating that it is suitable for factor analysis. We excluded the items if 1) items had load values less than 0.4 in the factor, 2) items had high load values and similar values within multiple factors, and 3) items could not explain the factor to which they belong. Through exploratory factor analysis, seven items were extracted. Factor 1 matched the “limitations on physical activities” dimension, and Factor 3 matched the “diet restrictions” dimension. Factor 2 and Factor 4, except 4i, were combined into the “hassles and burdens” dimension. Factor 5 and Factor 6 were combined, which were consistent with the “positive psychological impacts” dimension. We deleted Factor 7 since there remained only one item in this factor. The cumulative variance contribution rate was 73.30%, which was larger than 50%. The factor load coefficients are shown in Table 2.

**TABLE 2:** Factor load coefficients for items of Chinese DASS.

Dimension	Item	Factor 1	Factor 2	Factor 3	Factor 4	Factor 5	Factor 6	Factor 7
Limitations on physical activities	1a	0.855						
	1b	0.884						
	1c	0.633						
	1d	0.772						
	1e	0.666						
Diet restrictions	2a			0.758				
	2b			0.729				
	2c			0.759				
	2d			0.564				
Hassles and burdens	3a				0.870			
	3b				0.844			
	3c		0.609					
	3d		0.732					
	3e		0.466					
	3f		0.623					
	3g		0.629					
	3h		0.403					
	4i		0.666					
Positive psychological impacts	4a						0.824	
	4f						0.840	
	4j						0.645	
	4b					0.823		
	4d					0.439		
	4h					0.831		
	4g							0.884

Note: Factor load coefficients <0.4 are not shown in the table.

#### Option Selection Rate, CV, Critical Ratio, and Correlation Coefficient

As shown in Table 3, the selection rate of all options was lower than 80%, indicating a good ability to distinguish items in the scale. The CV values of all items were greater than 0.2, revealing good sensitivity of the items. T values were all greater than 3 in the high group (top 27%) and the low group (bottom 27%), which indicates a good critical ratio. Except for item 4f, the correlation coefficient between the scores of each item and the total score of the scale was larger than 0.4 (*p* < .001).

**TABLE 3:** Item screening of the Chinese version of DASS.

Item	No					CV	t	Alpha coefficient with the item removed	r	*p*
	5	4	3	2	1					
1a	119	62	6	2	0	0.43	−11.149	0.890	0.705	<.001
1b	120	60	9	0	0	0.41	−10.846	0.891	0.673	<.001
1c	88	80	17	3	1	0.45	−6.600	0.894	0.526	<.001
1d	107	74	7	1	0	0.40	−11.674	0.891	0.680	<.001
1e	91	86	11	1	0	0.39	−9.285	0.891	0.655	<.001
2a	63	119	5	2	0	0.33	−5.999	0.896	0.407	<.001
2b	59	118	9	3	0	0.34	−6.186	0.894	0.495	<.001
2c	50	120	12	4	3	0.39	−5.857	0.893	0.545	<.001
2d	86	92	11	0	0	0.37	−10.398	0.891	0.686	<.001
3a	106	71	12	0	0	0.41	−11.380	0.892	0.601	<.001
3b	108	69	12	0	0	0.41	−12.429	0.892	0.611	<.001
3c	106	73	6	4	0	0.44	−10.368	0.891	0.654	<.001
3d	88	93	8	0	0	0.36	−7.834	0.894	0.547	<.001
3e	136	46	7	0	0	0.41	−11.504	0.891	0.672	<.001
3f	100	84	5	0	0	0.37	−9.286	0.893	0.576	<.001
3g	68	103	18	0	0	0.36	−8.358	0.892	0.621	<.001
3h	77	89	15	8	0	0.44	−9.622	0.891	0.646	<.001
4a	11	68	72	35	3	0.32	−5.842	0.896	0.470	<.001
4b	72	101	7	9	0	0.42	−7.910	0.893	0.569	<.001
4d	36	122	26	5	0	0.33	−6.554	0.893	0.583	<.001
4f	80	88	16	5	0	0.58	−4.406	0.914	0.321	<.001
4h	80	88	16	5	0	0.43	−10.532	0.891	0.646	<.001
4i	82	98	7	2	0	0.38	−9.779	0.891	0.650	<.001
4j	35	55	68	31	0	0.39	−8.015	0.897	0.470	<.001

Note: CV, coefficient of variation.

#### The Alpha Coefficient of the Deleted Items

Cronbach’s α coefficient increased from 0.90 to 0.91 after excluding the 4f item. Therefore, we deleted 4f items and constructed the Chinese version of the DASS with four dimensions and 23 items.

#### Reliability and Validity

Cronbach’s α coefficient of the Chinese version of the DASS is 0.89. Among them, Cronbach’s α coefficients of four dimensions of limitations on physical activities, diet restrictions, hassles and burdens, positive psychological impacts are 0.89, 0.81, 0.89, and 0.74, respectively, revealing good internal consistency of the scale. The split-half reliability of this scale is 0.747 (>0.7), indicating good reliability of the scale. Confirmatory factor analysis showed acceptable fit of the model to the data (CMIN/DF = 1.825 < 5, GFI = 0.854 > 0.85, CFI = 0.938 > 0.9, RMSEA = 0.066 < 0.08, NFI = 0.875 < 0.9, TLI = 0.921 > 0.9).

Subgroup analysis was performed to validate the reliability of the Chinese version of the DASS among patients who were taking NOACs. Cronbach’s α coefficient of patients with NOACs was 0.90, with Cronbach’s α values of 0.90, 0.76, 0.88, and 0.73 for limitations on physical activities, diet restrictions, hassles and burdens, and positive psychological impacts, respectively, indicating good reliability of our scale for this group.

#### The Final Chinese Version of the DASS and Response Distribution for Each Item

**FIGURE 1:**
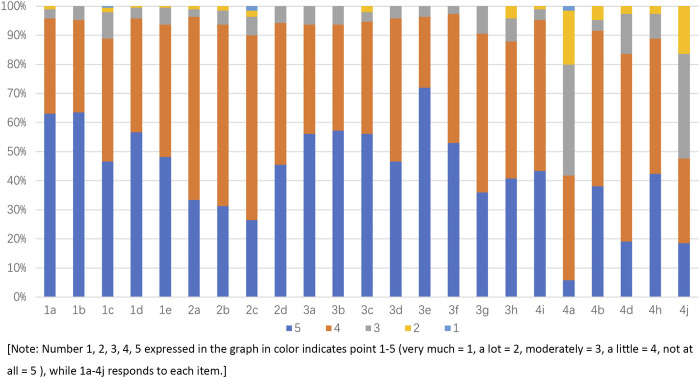
Response to each item of the Chinese version of the DASS.

After translation, back-translation, cultural adaption, item screening, and evaluation for reliability and validity, the final Chinese version of the DASS was formed as shown in Supplementary Table S2. The score of the DASS ranged from 66 to 115. A higher score means better quality of life and greater satisfaction. The mean score of the DASS was 98.72 ± 9.14 (mean ± SD), indicating good quality of life, which is shown in Figure 1.

## Discussion

Our study transculturally adapted and validated the Chinese version of the DASS and tested a group of 189 Chinese patients in a physician–pharmacist collaborative anticoagulation clinic to evaluate the satisfaction and quality of life of Chinese anticoagulation patients. This is the first study that applied the DASS to a Chinese population with good internal reliability and validity.

The process of DASS translation, transcultural adaptation, and item screening followed a standardized and widely accepted operation procedure based on the method proposed by Guillemin et al. (1993). The Chinese version of the DASS conducted in this study was comparable to the expression of the original version of the DASS (Samsa et al., 2004). We deleted two items (“4f: How much of an overall positive impact has anticoagulant therapy had on your life?” and “4g: How much of an overall negative impact has anticoagulant therapy had on your life?”). In addition, there was an item adapted from dimension 4 to dimension 3 (“4i: How difficult is it to cope with anticoagulant therapy compared to other treatments you have received?”). In addition, some expressions have been modified, such as “masseur” to “chiropractor”, to make them in line with Chinese cultural and linguistic characteristics.

The Chinese version of the DASS showed good reliability and validity comparable with the original English version (Samsa et al., 2004). In terms of reliability evaluation, Cronbach’s α coefficient of the Chinese version of the DASS was 0.89, which was no less than previous studies (English version 0.88 (Samsa et al., 2004), Brazilian Portuguese version 0.79 (Pelegrino et al., 2012), Maltese version 0.87 (Riva et al., 2019), and Arabic version 0.88 (AlAmmari et al., 2020)), indicating good reliability. In the Chinese version, Cronbach’s α coefficients of the dimension of limitations on physical activities, diet restrictions, and hassles and burdens were all above 0.8, whereas Cronbach’s α coefficient of positive psychological impacts was 0.74 and slightly lower than the other three dimensions. A relatively low Cronbach’s α coefficient of positive psychological impacts was also reported in the Brazilian–Portuguese version (0.67) (Pelegrino et al., 2012), Maltese version (0.65) (Riva et al., 2019), and the original English version (0.78) (Samsa et al., 2004). The lower figure for the positive impact dimension might result from the addition of more items and the construction of broader and more subjects for individual interpretation (Samsa et al., 2004). In terms of validity evaluation, there were minor discrepancies between the Chinese version and the original English version (Samsa et al., 2004) in several items. After item screening, AMOS 23.0 was used to conduct confirmatory factor analysis, which showed that the parameters of each fitting index were within the acceptable range, indicating that the model fit the survey data well, that the model was scientific and effective, and that the validity evaluation was good.

Considering the large population in China, the adaptation of the DASS was of vital importance. The weighted atrial fibrillation prevalence was 1.8%, with an estimated population of 7.9 million people in China (Du et al., 2021). The population-wide estimates of venous thromboembolism rates in Asia reported annual incidences of 13.8–19.9 per 100,000, which indicated more than 1 million patients in China every year (Lee et al., 2017). Consequently, there was supposed to be a large population on anticoagulation therapy in China. Studies (Balkhi et al., 2018; Bartoli-Abdou et al., 2018) have shown that anticoagulant treatment satisfaction was associated with INR control and adherence. The DASS, a scale specifically targeting at patients taking oral anticoagulants, was verified by different language versions (Pelegrino et al., 2012; Riva et al., 2019; AlAmmari et al., 2020). The introduction of this scale made contributions to the evaluation of satisfaction and quality of life in patients who were taking oral anticoagulants in China. The Chinese version of DASS helped identify limitations on physical activities and diet restrictions, and hassles and burdens to facilitate anticoagulation therapy and improve medication adherence. Patient education could be provided specifically by physicians and pharmacists to address the negative impacts of anticoagulation and to release related worries. At the same time, quality of life can also be used as one of the indicators to evaluate oral anticoagulant use.

The original DASS was developed early in 2004 when NOACs had not appeared on the market. More recently, NOACs have been popular treatment options for thrombotic disease since they showed similar or better efficacy and safety compared to warfarin (Dong et al., 2021). NOAC treatment is associated with greater satisfaction than warfarin, which is largely attributed to a lower degree of treatment burden with NOAC treatment (Katerenchuk et al., 2021). It was reported that Chinese patients using NOACs may have better quality of life than those with warfarin treatment (Zhang et al., 2017; Guan et al., 2018). Our study included patients taking both warfarin and NOACs. Although NOACs differ greatly from warfarin and do not need regular blood tests, the Chinese version of the DASS has been proven to apply well to patients taking NOACs, with a Cronbach’s α coefficient of 0.90, showing good reliability in subgroup analysis. It is worth mentioning that the sample size of our study was limited to detect the difference in quality of life between patients taking warfarin and NOACs in Chinese patients. However, it could be interesting to determine the type of anticoagulants with less impact on quality of life in further studies.

This study has some limitations. First, the unequal probability sampling method may lead to selection bias. In addition, data were not homogeneously collected as questionnaires were filled out by patients or assistants, which may also lead to some selection bias. Second, patients using warfarin and NOACs in Chinese patients may differ in quality of life, but we only analyzed NOACs and did not compare the two groups considering the low number of warfarin users. Third, our systematic review showed that the DASS was the optimal scale for evaluating the quality of life of anticoagulant patients, which lacked comparisons with other classical scales. Fourth, as this Chinese version was conducted in clinics at a tertiary teaching hospital, the application of it across multiple models of anticoagulation, such as ambulatory care, needs to be validated.

## Conclusion

The Chinese version of the DASS with four dimensions and 25 items has shown levels of reliability and validity comparable with the original English version. It would provide a practical instrument for healthcare practitioners to assess satisfaction and quality of life among Chinese patients receiving oral anticoagulant therapy. The difference in quality of life between patients taking warfarin and NOACs needs to be further explored in future studies.

## Data Availability

The original contributions presented in the study are included in the article/Supplementary Material; further inquiries can be directed to the corresponding author.
