# Supplementary material for: The Transcultural Adaptation and Validation of the Chinese Version of the Duke Anticoagulation Satisfaction Scale
Source: Front Pharmacol. 2022 Feb 23;13:790293. doi: 10.3389/fphar.2022.790293 (PMC8904917; doi:10.3389/fphar.2022.790293)
Supplement: Supplementary file 1 [file Table1.DOCX]

**Supplementary Table 1.** The comparison between English and Chinese item.

| No. | English item | Chinese item |
| --- | --- | --- |
| 1a | How much does the possibility of bleeding or bruising limit you from taking part in physical activities (for example, housework, gardening, dancing, sports, or anything else you would usually do)? | 出血风险的增加对您的体力活动（例如，家务劳动，园艺工作，跳舞，运动等）产生了多大的限制？ |
| 1b | How much does the possibility of bleeding or bruising limit you from traveling? | 出血风险的增加对您的行程产生了多大的限制？ |
| 1c | How much does the possibility of bleeding or bruising limit you from getting the medical care you need (for example, visiting a dentist, chiropractor, or doctor of your choice)? | 出血风险的增加对您的医疗保健（例如，看牙医、推拿按摩师等）产生了多大的限制？ |
| 1d | How much does the possibility of bleeding or bruising limit your ability to work for pay? | 出血风险的增加对您的工作能力产生了多大的限制？ |
| 1e | Overall, how much does the possibility of bleeding or bruising affect your daily life? | 总体而言，出血风险的增加对您的日常生活有多大影响？ |
| 2a | How much does anti-clot treatment limit your choice of food (diet)? | 抗凝治疗对您饮食的选择产生了多大的限制？ |
| 2b | How much does anti-clot treatment limit the alcoholic beverages you might wish to drink? | 当您想要饮用酒精饮料时，抗凝治疗对您产生了多大的限制？ |
| 2c | How much does anti-clot treatment limit the over-the-counter medications (for example, aspirin, ibuprofen, vitamins) you might wish to take? | 当您需要服用非处方药(如阿司匹林、布洛芬和维生素)时，抗凝治疗对您产生了多大的限制？ |
| 2d | Overall, how much does anti-clot treatment affect your daily life? | 总体而言，抗凝治疗对您的日常生活产生了多大的限制？ |
| 3a | How much of a hassle (inconvenience) are the daily tasks of anti-clot treatment? | 抗凝治疗的日常工作给您造成了多大的麻烦？ |
| 3b | How much of a hassle (inconvenience) are the occasional tasks of anti-clot treatment? | 抗凝治疗的非日常工作给您造成了多大的麻烦？ |
| 3c | How much of a hassle (inconvenience) are the daily tasks of anti-clot treatment? | 您认为自己的抗凝治疗有多复杂？ |
| 3d | How time-consuming do you find your anti-clot treatment to be? | 您认为自己的抗凝治疗有多耗时？ |
| 3e | How frustrating do you find your anti-clot treatment to be? | 您认为自己的抗凝治疗有多令人沮丧？ |
| 3f | How painful do you find your anti-clot treatment to be? | 您认为自己的抗凝治疗有多痛苦？ |
| 3g | Overall, how much of a burden do you find your anti-clot treatment to be? | 总体而言，您认为抗凝治疗给您带来了多大的负担？ |
| 3h | Overall, how confident are you about handling your anti-clot treatment | 总体而言，您对抗凝治疗的成功有多少信心？ |
| 4a | How well do you feel that you understand the medical reason for your anti-clot treatment? | 您认为自己对抗凝治疗医学原理的了解有多少？ |
| 4b | How much do you feel reassured because of your anti-clot treatment? | 接受抗凝治疗在多大程度上使您更安心？ |
| 4d | How much do you worry about bleeding and bruising? | 您对流血或擦伤事件的发生有多担心？ |
| 4f | Overall, how much has anti-clot treatment had a positive impact on your life? | 总体而言，抗凝治疗对您生活产生的积极影响有多大？ |
| 4g | Overall, how much has anti-clot treatment had a negative impact on your life? | 总体而言，抗凝治疗对您生活产生的消极影响有多大？ |
| 4h | Overall, how satisfied are you with your anti-clot treatment? | 总体而言，您对自己抗凝治疗的满意度是多少？ |
| 4i | Compared with other treatments you have had, how difficult is your anti-clot treatment to manage? | 与您曾经接受的其他治疗相比，应对抗凝治疗的难度有多大？ |
| 4j | How likely would you be to recommend this form of anti-clot treatment to someone else with your disease or medical condition? | 您会向与您患有同样疾病或有相似情况的人推荐这种抗凝治疗吗？ |

*Note: The original questionnaire of the Chinese DASS includes 25 items distributed in limitations on physical activities (1a, 1b, 1c, 1d, 1e), diet restrictions (2a, 2b, 2c, 2d), hassles and burdens (3a, 3b, 3c, 3d, 3e, 3f, 3g, 3h), and positive psychological effect (4a, 4b, 4d, 4f, 4g, 4h, 4i, 4j).*
