# Supplementary material for: The Transcultural Adaptation and Validation of the Chinese Version of the Duke Anticoagulation Satisfaction Scale
Source: Front Pharmacol. 2022 Feb 23;13:790293. doi: 10.3389/fphar.2022.790293 (PMC8904917; doi:10.3389/fphar.2022.790293)
Supplement: Supplementary file 2 [file Table2.DOCX]

**Supplementary Table 2.** The final Chinese version of the DASS

这是一份抗凝治疗对您生活质量影响的评定量表（即抗凝治疗对您生活质量的影响以及您对抗凝治疗的了解和感受）。请选择最符合您情况的答案。当您从未有过该情况时，请选择“完全没有”。

**Limitations on physical activities**

服用抗凝药可能使您有增加出血或擦伤出血的风险，可能导致活动受到限制。“限制”意味着您减少该活动甚至不再参与。

| 序号 | 内容 | 选项 | | | | |
| --- | --- | --- | --- | --- | --- | --- |
| 1a | 出血风险的增加对您的体力活动（例如，家务劳动，园艺工作，跳舞，运动等）产生了多大的限制？ | 完全没有限制 | 不太限制 | 有点限制 | 较大限制 | 非常大限制 |
| 1b | 出血风险的增加对您的行程产生了多大的限制？ | 完全没有限制 | 不太限制 | 有点限制 | 较大限制 | 非常大限制 |
| 1c | 出血风险的增加对您的医疗保健（例如，看牙医、推拿按摩师等）产生了多大的限制？ | 完全没有限制 | 不太限制 | 有点限制 | 较大限制 | 非常大限制 |
| 1d | 出血风险的增加对您的工作能力产生了多大的限制？ | 完全没有限制 | 不太限制 | 有点限制 | 较大限制 | 非常大限制 |
| 1e | 总体而言，出血风险的增加对您的日常生活有多大影响？ | 完全没有影响 | 不太影响 | 有点影响 | 较大影响 | 非常大影响 |

**Diet restrictions**

接受抗凝治疗可能也会改变您的其他习惯。

| 序号 | 内容 | 选项 | | | | |
| --- | --- | --- | --- | --- | --- | --- |
| 2a | 抗凝治疗对您饮食的选择产生了多大的限制？ | 完全没有限制 | 不太限制 | 有点限制 | 较大限制 | 非常大限制 |
| 2b | 当您想要饮用酒精饮料时，抗凝治疗对您产生了多大的限制？ | 完全没有限制 | 不太限制 | 有点限制 | 较大限制 | 非常大限制 |
| 2c | 当您需要服用非处方药(如阿司匹林、布洛芬和维生素)时，抗凝治疗对您产生了多大的限制？ | 完全没有限制 | 不太限制 | 有点限制 | 较大限制 | 非常大限制 |
| 2d | 总体而言，抗凝治疗对您的日常生活产生了多大的限制？ | 完全没有限制 | 不太限制 | 有点限制 | 较大限制 | 非常大限制 |

**Hassles and burdens**

接受抗凝治疗意味着做很多事情，有些需要每天都做，有些只是需要患者偶尔去做。

日常管理包括：按时服药，服用正确剂量的药品，限制饮酒，合理饮食，避免擦伤和流血等。

非日常管理包括：前往诊所进行血液检查，在发生出血或其他不良反应时与诊所联系等。

| 序号 | 内容 | 选项 | | | | |
| --- | --- | --- | --- | --- | --- | --- |
| 3a | 抗凝治疗的日常工作给您造成了多大的麻烦？ | 完全不麻烦 | 不太麻烦 | 有点麻烦 | 比较麻烦 | 非常麻烦 |
| 3b | 抗凝治疗的非日常工作给您造成了多大的麻烦？ | 完全不麻烦 | 不太麻烦 | 有点麻烦 | 比较麻烦 | 非常麻烦 |
| 3c | 您认为自己的抗凝治疗有多复杂？ | 完全不复杂 | 不太复杂 | 有点复杂 | 比较复杂 | 非常复杂 |
| 3d | 您认为自己的抗凝治疗有多耗时？ | 完全不耗时 | 不太耗时 | 有点耗时 | 比较耗时 | 非常耗时 |
| 3e | 您认为自己的抗凝治疗有多令人沮丧？ | 完全不沮丧 | 不太沮丧 | 有点沮丧 | 比较沮丧 | 非常沮丧 |
| 3f | 您认为自己的抗凝治疗有多痛苦？ | 完全不痛苦 | 不太痛苦 | 有点痛苦 | 比较痛苦 | 非常痛苦 |
| 3g | 总体而言，您认为抗凝治疗给您带来了多大的负担？ | 完全没有负担 | 不太有负担 | 有点负担 | 较大负担 | 非常大负担 |
| 3h | 总体而言，您对抗凝治疗的成功有多少信心？ | 非常有信心 | 比较有信心 | 有点信心 | 不太有信心 | 完全没有信心 |
| 4i | 与您曾经接受的其他治疗相比，应对抗凝治疗的难度有多大？ | 完全没有难度 | 不太有难度 | 有点难度 | 难度比较大 | 难度非常大 |

**Positive psychological effect**

最后这些问题是关于您对抗凝治疗的了解和看法。

| 序号 | 内容 | 选项 | | | | |
| --- | --- | --- | --- | --- | --- | --- |
| 4a | 您认为自己对抗凝治疗医学原理的了解有多少？ | 非常了解 | 比较了解 | 了解一点 | 不太了解 | 完全不了解 |
| 4b | 接受抗凝治疗在多大程度上使您更安心？ | 非常安心 | 比较安心 | 有点安心 | 不太安心 | 完全不安心 |
| 4d | 您对流血或擦伤事件的发生有多担心？ | 完全不担心 | 不太担心 | 有点担心 | 比较担心 | 非常担心 |
| 4h | 总体而言，您对自己抗凝治疗的满意度是多少？ | 非常满意 | 比较满意 | 一般满意 | 不太满意 | 完全不满意 |
| 4j | 您会向与您患有同样疾病或有相似情况的人推荐这种抗凝治疗吗？ | 一定会 | 很大可能会 | 可能会 | 不太会 | 完全不会 |
